# Racial/ethnic disparities in renal cell carcinoma: Increased risk of early‐onset and variation in histologic subtypes

**DOI:** 10.1002/cam4.2552

**Published:** 2019-09-11

**Authors:** Ken Batai, Alfredo Harb‐De la Rosa, Jiping Zeng, Juan J. Chipollini, Francine C. Gachupin, Benjamin R. Lee

**Affiliations:** ^1^ Department of Urology University of Arizona Tucson Arizona; ^2^ Department of Family and Community Medicine University of Arizona Tucson Arizona

**Keywords:** American Indians, early onset, health disparities, Hispanic Americans, renal cell carcinoma subtypes

## Abstract

**Background:**

Racial/ethnic minority groups have a higher burden of renal cell carcinoma (RCC), but RCC among Hispanic Americans (HAs) and American Indians and Alaska Natives (AIs/ANs) are clinically not well characterized. We explored variations in age at diagnosis and frequencies of RCC histologic subtypes across racial/ethnic groups and Hispanic subgroups using National Cancer Database (NCDB) and Arizona Cancer Registry Data.

**Methods:**

Adult RCC cases with known race/ethnicity were included. Logistic regression analysis was performed to estimate odds and 95% confidence interval (CI) of early‐onset (age at diagnosis <50 years) and diagnosis with clear cell RCC (ccRCC) or papillary RCC.

**Results:**

A total of 405 073 RCC cases from NCDB and 9751 cases from ACR were identified and included. In both datasets, patients from racial/ethnic minority groups had a younger age at diagnosis than non‐Hispanic White (NHW) patients. In the NCDB, AIs/ANs had twofold increased odds (OR, 2.21; 95% CI, 1.88‐2.59) of early‐onset RCC compared with NHWs. HAs also had twofold increased odds of early‐onset RCC (OR, 2.14; 95% CI, 1.79‐2.55) in the ACR. In NCDB, ccRCC was more prevalent in AIs (86.3%) and Mexican Americans (83.5%) than NHWs (72.5%). AIs/ANs had twofold increased odds of diagnosis with ccRCC (OR, 2.18; 95% CI, 1.85‐2.58) in the NCDB, but the association was stronger in the ACR (OR, 2.83; 95% CI, 2.08‐3.85). Similarly, Mexican Americans had significantly increased odds of diagnosis with ccRCC (OR, 2.00; 95% CI, 1.78‐2.23) in the NCDB.

**Conclusions:**

This study reports younger age at diagnosis and higher frequencies of ccRCC histologic subtype in AIs/ANs and Hispanic subgroups. These variations across racial/ethnic groups and Hispanic subgroups may have potential clinical implications.

## Graphical abstract

Renal cell carcinoma patients from racial/ethnic minority groups have increased risk of diagnosis at a younger age than non‐Hispanic White patients

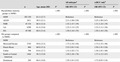

## 1 INTRODUCTION

Kidney cancer incidence and mortality rates in the United States (US) vary among the different racial/ethnic groups, and kidney cancer incidence and mortality rates are higher in American Indians and Alaska Natives (AIs/ANs) and non‐Hispanic Blacks (NHBs) compared with non‐Hispanic Whites (NHWs).^1^, ^2^, ^3^, ^4^, ^5^ Hispanic Americans (HAs) in the US‐Mexico border states also have higher kidney cancer rates than NHW,^6^ and the US‐born HAs in Texas and California have higher kidney cancer mortality rate compared with NHWs.^7^

Many kidney cancer health disparities studies have explored clinical and pathological characteristics and causes of health disparities in NHBs.^8^, ^9^, ^10^, ^11^, ^12^ Difference in prevalence of renal cell carcinoma (RCC) histologic subtypes and age of diagnosis between NHBs and NHWs has been recognized. Studies have shown that compared to NHW papillary RCC is more common, and clear cell RCC (ccRCC) is less common in NHBs.^13^, ^14^ NHBs also have a younger age of diagnosis compared with NHWs.^11^, ^12^ A study of Surveillance, Epidemiology, and End Results data showed NHB and AIs/ANs had a younger age of diagnosis than NHWs.^15^ However, AIs/ANs and HAs are underrepresented in clinical and epidemiologic studies of kidney cancer, and clinical and pathological characteristics as well as the pattern of health disparities among them are not well understood.^6^ Our previous study of RCC demonstrated that HAs and AIs from southern Arizona were diagnosed with RCC at a younger age than NHWs, and ccRCC was more common among HAs and AIs than NHWs.^16^ A study showed that HA RCC patients from northern California had a significantly lower median age at diagnosis than NHWs, and ccRCC was slightly more common in HAs than NHW. Another study from Texas showed that ccRCC was very common among HAs, but sample size was small.^17^ These studies of HAs were conducted with patient data from a single institution or health care system. There is no study showing prevalence of histologic subtypes in AIs/ANs.

In order to validate our findings, we explored variation in age at diagnosis and frequencies of RCC histologic subtypes across racial/ethnic groups and Hispanic subgroups at national as well as state levels using data obtained from National Cancer Database (NCDB) and Arizona Cancer Registry (ACR). This study focuses on 1) age at diagnosis because of a greater increase in RCC incidence among the younger age groups 18 and 2) histologic subtypes because of a difference in prognosis^19^, ^20^ and treatment response.^21^ Moreover, because cancer incidence and mortality rates vary across US and AIs/ANs and HAs are heterogeneous groups, it is necessary to examine variation in clinical and pathological characteristics at many different levels (eg, national and state levels) to understand the pattern of RCC health disparities.

## 2 MATERIALS AND METHODS

### 2.1 Renal cell carcinoma case data

De‐identified data of kidney cancer (ICD‐10 Code C649) patients were obtained from the NCDB and ACR. Of all kidney cancer cases, we included only adult RCC cases (age at diagnosis ≥18 years and ≤90 years) for this study, which include 408 529 RCC patients diagnosed between 2004 and 2015 obtained from the NCDB and 9975 cases diagnosed between 2007 and 2016 obtained from the ACR. This study considered diagnosis of RCC with age <50 as early‐onset, because patients with Birt‐Hogg‐Dube syndrome had median age of diagnosis at age 50,^15^ while patients with other hereditary syndromes have a younger median age at diagnosis. This suggest that patients who are diagnosed before age 50 have high potential of having hereditary RCC.

The NCDB is one of the largest cancer registries and has hospital based clinical data reported from more than 1500 American College of Surgeon Commission on Cancer accredited hospitals.^22^, ^23^ The NCDB captures over 70% of cancer cases diagnosed in the US The NCDB has data neighborhood socioeconomic characteristics (derived from matching the zip code of the patient record to 2000 US Census data), hospitals types (Community Cancer Program, Comprehensive Community Cancer Program, Academic/Research Program, or Integrated Network Cancer Program), geographic region, geographic distance to health care center, patient's demographic and clinical characteristics (age, race/ethnicity, gender, tumor histology, stage at diagnosis, first course therapy, and type of surgical resection). The NCDB has Charlson/Dayo Score (0, 1, and 2). If patient has myocardial infarction, congestive heart failure, peripheral vascular disease, cerebrovascular disease, dementia, chronic pulmonary disease, rheumatologic disease, peptic ulcer disease, mild liver disease, or diabetes, score of 1 is given. If the patient has diabetes with chronic complications, hemiplegia or paraplegia, renal disease, moderate or severe liver disease, or HIV, score of 2 is given. Although the NCDB is the largest registry, it has inherent bias. The data includes cases from health care systems that tend to be larger in size, and it has low coverages for AIs/ANs and HAs as well as kidney cancer cases in the Mountain and Pacific states, especially Arizona.^24^, ^25^

The ACR is a population‐based surveillance system. The ACR data include AI kidney cancer cases reported from the Indian Health Service (IHS) facilities in Arizona, that are not included in the NCDB. We obtained basic demographic (age at diagnosis, race/ethnicity, gender, marital status) and pathologic (tumor grade, stage at diagnosis, and histologic subtype) information. The ACR has information on whether the cases come from IHS. Because AIs are often misclassified as other racial/ethnic groups,^26^, ^27^ this information can be used to identify miss‐classified AI cases. The ACR also has information on HA subgroups and if the patients were born in the US or other countries. Both ACR and NCDB use American Join Committee on Cancer TNM staging system.

In this study, patients with unknown race/ethnic identity and patients who did not identify themselves as NHW, AI/AN, HA, NHB, or Asian American (AA) were excluded. Among HAs in the NCDB dataset, only patients with known origin, Mexican/Chicano, Puerto Rican, Cuban, South or Central American, and Dominican were included for subgroup analysis. We focused on HAs of Mexican origin in the ACR dataset, because 90.2% of HAs with known origin were Mexican Americans and HAs of other origin were not well represented. Instead, we further stratified Mexican Americans whether they were US‐born or Mexico‐born. AIs from the ACR include cases classified as other racial/ethnic groups but reported from the IHS facilities. Cases with known histologic subtypes (histologic code, 8255, 8260, 8310, 8316, 8317, 8318, and 8319) were included, while patients with unknown RCC histologic subtype (not otherwise specified, NOS) were excluded.

### 2.2 Statistical analysis

One‐way ANOVA and Chi‐squared test were used to examine the difference in age at diagnosis and histological subtypes across racial/ethnic groups. Independent sample T‐test was also used to compare age at diagnosis between NHWs and racial/ethnic minority groups. Logistic regression analysis was performed to assess odds of early‐onset RCC and diagnosis with ccRCC and papillary RCC. Odds ratio (OR) and 95% confidence interval (CI) of early‐onset of RCC (age at diagnosis <50 years) was estimated using regression model adjusting for gender, facility type, Charlson/Deyo Score, RCC histologic subtype, stage at diagnosis, and insurance type for the NCDB and adjusting for gender, marital status, stage at diagnosis, and RCC histologic subtype for the ACR. Logistic regression analysis to test association between histologic subtypes and race/ethnicity was performed adjusting for age (categorical), gender, Charlson‐Deyo Score, and facility type for the NCDB and adjusting for age and gender for the ACR data. During the initial model building process, other variables were included in the regression models. In our final models, we excluded variables that were not associated with early‐onset or RCC subtypes and if exclusion of them did not change ORs for the race/ethnicity variable by 10% or more.

## 3 RESULTS

### 3.1 National Cancer Database

A total of 405 073 RCC cases from the NCDB were included (Table 1). Racial/ethnic minority patients were diagnosed at a younger age than NHW patients. AIs/ANs had the lowest mean age at diagnosis (58.5 years in AIs/ANs vs 63.2 years in NHWs), and 23.1% of AIs/ANs were diagnosed before age 50 years. AIs/ANs were also more likely to be treated at community cancer programs and have public health insurance and comorbidities (Table S1). Among HAs, South and Central Americans had the lowest mean age at diagnosis (58.3 years). Mexican Americans also had low mean age at diagnosis (59.0 years), and 24.0% of them were diagnosed before age 50 years. Mexican Americans were often treated at community health programs and have advanced stage RCC (Stage III or IV, Table S2). Puerto Ricans were more likely to use public insurance and have more comorbidities. High proportions of Mexican Americans and South or Central American were uninsured compared to NHWs.

**Table 1:** Association between race/ethnicity and early‐onset RCC (<50 years old) in National Cancer Database (n = 405 073)

	n	Age, mean (SD)	*P*	All subtypes^a^		ccRCC only^b^	
				OR (95% CI)	*P*	OR (95% CI)	*P*
Racial/ethnic minority groups compared to NHWs			<.001		<.001		<.001
NHW	302 230	63.2 (12.7)		Reference		Reference	
AI/AN	1811	58.5 (12.1)		2.21 (1.88‐2.59)		2.25 (1.89‐2.67)	
NHB	45 334	60.4 (12.4)		1.52 (1.47‐1.59)		1.31 (1.24‐1.39)	
AA	6390	61.4 (13.6)		1.23 (1.12‐1.35)		1.23 (1.11‐1.36)	
HA	49 308	60.4 (13.4)		1.50 (1.44‐1.55)		1.50 (1.44‐1.56)	
HA groups compared to NHWs			<.001		<.001		<.001
NHW				Reference		Reference	
Mexican/Chicano	3745	59.0 (13.2)		1.73 (1.54‐1.94)		1.73 (1.53‐1.96)	
Puerto Rican	866	60.0 (13.2)		2.52 (2.01‐3.16)		2.50 (1.91‐3.26)	
Cuban	798	63.6 (12.7)		1.32 (1.02‐1.71)		1.38 (1.01‐1.88)	
South or Central American	1464	58.3 (13.0)		1.50 (1.26‐1.78)		1.37 (1.12‐1.67)	
Dominican	269	60.4 (14.0)		1.59 (1.01‐2.53)		1.72 (0.93‐3.18)	

Abbreviations: AA, Asian American; AI/AN, American Indian and Alaska Native; ccRCC, clear cell renal cell carcinoma; HA, Hispanic American; NHB, Non‐Hispanic Black; NHW, Non‐Hispanic White; RCC, renal cell carcinoma.

a Early‐onset (age < 50) compared to late‐onset (≥age 50) adjusting for gender, facility type, Charlson/Deyo Score, RCC histologic subtype, stage at diagnosis, and insurance type.

b Early‐onset (age < 50) compared to late‐onset (≥age 50) adjusting for gender, facility type, Charlson/Deyo Score, stage at diagnosis, and insurance type.

Compared to NHWs, we observed significantly increased odds of early‐onset RCC in NHBs, HAs, AIs/ANs, and AAs (Table 1). Odd of early‐onset was greatest in AIs/ANs, and AIs/ANs had over twofold increased odds of early‐onset (OR, 2.21; 95% CI, 1.88‐2.59). Increased odds of early‐onset RCC compared to NHWs were observed for all the Hispanic subgroups analyzed. The analysis including only ccRCC cases revealed similar results. Additional adjustment for neighborhood characteristics (urban/rural 2013, % no high school degree 2008‐2012, and median income quartiles 2008‐2012) and year of diagnosis did not change the results.

ccRCC was more common in AIs/ANs (86.3%) and Mexican Americans (83.5%) than in NHWs (72.5%, Table 2). AIs/ANs had twofold increased odds of diagnosis with ccRCC compared to NHWs (OR, 2.18; 95% CI, 1.85‐2.58). AAs and HAs also had increased odds of diagnosis with ccRCC. Among HAs, Mexican Americans and South or Central Americans had increased odds of diagnosis with ccRCC (OR, 2.00; 95% CI, 1.78‐2.23 and OR, 1.37; 95% CI, 1.17‐1.60 respectively). ccRCC was not very common in NHBs (43.9%) and Dominicans (52.0%), and NHBs, Dominicans, and also Cubans had significantly reduced odds of diagnosis with ccRCC. Papillary RCC was more common in NHBs (40%) and Dominicans (24.3%) than NHWs (15.0%). Compared with NHWs, NWBs had fourfold and Dominicans had almost twofold increased odds of diagnosis with papillary RCC. Papillary RCC was not very common in AIs/ANs, AAs, and HAs, particularly Mexican Americans and South or Central Americans, and they had significantly reduced odds of diagnosis with papillary RCC.

**Table 2:** Association between race/ethnicity and RCC histologic subtypes in National Cancer Database

	Clear Cell	Papillary	Chromophobe	Other	*P*	Clear Cell RCC^a^	*P*	Papillary RCC^b^	*P*
						OR (95% CI)		OR (95% CI)	
Racial/ethnic minority groups compared to NHWs					<.001		<.001		<.001
NHW	147 523 (72.5)	30 608 (15.0)	13 850 (6.8)	11 408 (5.6)		Reference		Reference	
AI/AN	1077 (86.3)	68 (5.4)	41 (3.3)	62 (5.0)		2.18 (1.85‐2.58)		0.37 (0.29‐0.48)	
NHB	12 876 (43.9)	11 749 (40.0)	2317 (7.9)	2418 (8.2)		0.29 (0.28‐0.29)		4.01 (3.90‐4.13)	
AA	3585 (77.4)	481 (10.4)	312 (6.7)	253 (5.5)		1.39 (1.30‐1.50)		0.62 (0.56‐0.69)	
HA	23 570 (74.4)	3924 (12.4)	2369 (7.5)	1817 (5.7)		1.08 (1.05‐1.11)		0.85 (0.81‐0.88)	
HA groups compared to NHWs					<.001		<.001		<.001
NHW						Reference		Reference	
Mexican/Chicano	2126 (83.5)	153 (6.0)	145 (5.7)	122 (4.8)		2.00 (1.78‐2.23)		0.38 (0.32‐0.45)	
Puerto Rican	423 (70.4)	85 (14.1)	60 (10.0)	33 (5.5)		0.89 (0.74‐1.07)		1.01 (0.80‐1.29)	
Cuban	345 (66.7)	79 (15.3)	46 (8.9)	47 (9.1)		0.78 (0.65‐0.94)		1.01 (0.79‐1.29)	
South or Central American	807 (77.7)	87 (8.4)	91 (8.8)	54 (5.2)		1.37 (1.17‐1.60)		0.50 (0.40‐0.64)	
Dominican	92 (52.0)	43 (24.3)	26 (14.7)	16 (9.0)		0.39 (0.29‐0.53)		1.98 (1.38‐2.84)	

Abbreviations: AA, Asian American; AI/AN, American Indian and Alaska Native; HA, Hispanic American; NHB, Non‐Hispanic Black; NHW, Non‐Hispanic White; RCC, renal cell carcinoma.

a Clear cell RCC compared to other subtypes adjusting for age, gender, Charlson/Deyo Score, and facility type.

b Papillary RCC compared to other subtypes adjusting for age, gender, Charlson/Deyo Score, and facility type.

### 3.2 Arizona cancer registry

A total of 9751 RCC cases from ACR was included in this analysis (Table 3). RCC patients from racial/ethnic minority groups in Arizona were diagnosed at younger age than NHW patients as in the NCDB, and AI patients had the lowest mean age at diagnosis (58.9 years in AIs vs. 64.3 years in NHWs). The US‐born Mexican Americans had similar mean age of diagnosis as NHWs, while Mexico‐born Americans (mean age 61.7 years) were diagnosed at a younger age than NHWs. The difference between US‐born and Mexico‐born Mexican Americans was not statistically significant. Compared to NHWs, HAs, AIs, and NHBs were more likely to be single at the time of diagnosis (Table S3). Both US‐born and Mexico‐born Mexican Americans were often diagnosed with advanced stage RCC (Table S4).

**Table 3:** Association between race/ethnicity and early‐onset RCC (<50 years old) in Arizona Cancer Registry (n = 9751)

	n	Age (SD)	*P*	All subtypes^a^		ccRCC only^b^	
				OR (95% CI)	*P*	OR (95% CI)	*P*
Racial/ethnic minority groups compared to NHWs			<.001		<.001		<.001
NHW	6965	64.3 (12.6)		Reference		Reference	
AI	632	58.9 (12.9)		2.17 (1.68‐2.81)		2.05 (1.55‐2.70)	
NHB	330	59.9 (13.3)		1.79 (1.23‐2.62)		1.31 (0.71‐2.41)	
AA	84	61.2 (14.3)		2.09 (0.99‐4.38)		2.19 (0.94‐5.09)	
HA	1740	59.3 (13.2)		2.14 (1.79‐2.55)		2.05 (1.69‐2.48)	
Mexican Americans compared to NHWs			<.001		<.001		.01
NHW				Reference		Reference	
Mexican	734	62.0 (13.2)		1.69 (1.27‐2.24)		1.50 (1.10‐2.05)	
US/Mexico‐born Mexican Americans compared to NHWs			.004		.07		.17
NHW				Reference		Reference	
US‐Born Mexican	335	63.5 (13.5)		1.39 (0.90‐2.14)		1.20 (0.74‐1.96)	
Mexico‐Born	238	61.7 (12.3)		1.57 (0.98‐2.52)		1.59 (0.96‐2.65)	

Abbreviations: AA, Asian American; AI/AN, American Indian and Alaska Native; ccRCC, clear cell renal cell carcinoma; HA, Hispanic American; NHB, Non‐Hispanic Black; NHW, Non‐Hispanic White; RCC, renal cell carcinoma.

a Early‐onset (age < 50) compared to late‐onset (≥age 50) adjusting for gender, marital status, stage at diagnosis, and RCC histologic subtype.

b Early‐onset (age < 50) compared to late‐onset (≥age 50) adjusting for gender, marital status, and stage at diagnosis.

Odds of early‐onset RCC were greater for racial/ethnic minority groups. HAs had twofold increased odds of early‐onset RCC compared to NHWs (OR, 2.14; 95%CI, 1.79‐2.55), and AIs had twofold increased odds of early‐onset ccRCC compared to NHWs (OR, 2.17; 95% CI, 1.68‐2.81). Analysis only for ccRCC revealed similar results. When we compared Mexican American with NHWs, Mexican Americans had significantly increased odds of early diagnosis. However, we did not observe significant association, when Mexican Americans were separated into US‐born and Mexico‐born.

ccRCC was more common in AIs (88.7%) and HAs (84.0%) than NHWs (72.4%) (Table 4). AIs had almost threefold increased odds (OR, 2.83; 95% CI, 2.08‐3.85) and HAs had almost twofold increased odds (OR, 1.90; 95% CI, 1.60‐2.25) of diagnosis with ccRCC compared to NHWs. US‐born and Mexico‐born Mexican Americans had very similar frequencies of ccRCC. US‐born and Mexico‐born Mexican Americans had similar odds of diagnosis with ccRCC compared with NHWs, and Mexican Americans had increased odds of diagnosis of ccRCC compared with NHWs. ccRCC was less common in NHBs (41.9%), and NHBs had significantly reduced odds of ccRCC diagnosis (OR, 0.26; 95% CI, 0.20‐0.35). Papillary RCC was more common in NHBs (38.9%) than NHWs (13.2%). NHBs had more than fourfold increased odds of diagnosis with papillary RCC (OR, 4.52; 95% CI, 3.40‐6.03), while AIs and HAs had reduced odds of diagnosis with papillary RCC.

**Table 4:** Association between race/ethnicity and RCC histologic subtypes in Arizona Cancer Registry

	Clear Cell	Papillary	Chromophobe	Other	*P*	Clear Cell RCC^a^		Papillary RCC^b^	
						OR (95% CI)	*P*	OR (95% CI)	*P*
Racial/ethnic minority groups compared to NHWs					<.001		<.001		<.001
NHW	3641 (72.4)	664 (13.2)	460 (9.2)	262 (5.2)		Reference		Reference	
AI	377 (88.7)	22 (5.2)	8 (1.9)	18 (4.2)		2.83 (2.08‐3.85)		0.39 (0.25‐0.61)	
NHB	96 (41.9)	89 (38.9)	27 (11.8)	17 (7.4)		0.26 (0.20‐0.35)		4.52 (3.40‐6.03)	
AA	42 (68.9)	11 (18.0)	5 (8.2)	3 (4.9)		0.78 (0.45‐1.36)		1.65 (0.84‐3.21)	
HA	980 (84.0)	71 (6.1)	65 (5.6)	51 (4.4)		1.90 (1.60‐2.25)		0.46 (0.36‐0.60)	
Mexican Americans compared to NHWs					<.001		<.001		<.001
NHW						Reference		Reference	
Mexican	362 (82.5)	30 (6.8)	25 (5.7)	22 (5.0)		1.75 (1.35‐2.25)		0.51 (0.35‐0.74)	
US/Mexico‐born Mexican Americans compared to NHWs					.001		.001		.02
NHW						Reference		Reference	
US‐Born Mexican	160 (82.1)	16 (8.2)	7 (3.6)	12 (6.1)		1.76 (1.21‐2.55)		0.58 (0.34‐0.97)	
Mexico‐Born	123 (82.6)	10 (6.7)	7 (4.7)	9 (6.0)		1.73 (1.13‐2.66)		0.51 (0.27‐0.98)	

Abbreviations: AA, Asian American; AI/AN, American Indian and Alaska Native; ccRCC, clear cell renal cell carcinoma; HA, Hispanic American; NHB, Non‐Hispanic Black; NHW, Non‐Hispanic White; RCC, renal cell carcinoma.

a Diagnosis with clear cell RCC compared to other RCC histologic subtypes adjusting for age and gender.

b Diagnosis with papillary RCC compared to other RCC histologic subtypes adjusting for age and gender.

## 4 DISCUSSION

NHBs, AIs/ANs, and US‐born HAs have heavier burden of RCC with higher incidence and/or mortality rates than NHWs.^1^, ^7^ AIs/ANs have the highest kidney cancer incidence and mortality rates,^1^ and AIs/ANs have almost twofold increased kidney cancer mortality rate compared to NHWs.^3^ HAs from California and Texas also have 40%‐50% increased risk of kidney cancer mortality.^7^ Kidney cancer burden in NHBs has been previously recognized, and many studies explored kidney cancer health disparities focusing on NHBs.^8^, ^9^, ^10^, ^11^, ^12^ As a step toward better understanding of kidney cancer health disparities, this study explored the variation in age at diagnosis and histologic subtype across major racial/ethnic groups in the US as well as Hispanic subgroups and showed that RCC patients from racial/ethnic minority backgrounds are diagnosed at younger age and more likely to have different RCC subtypes compared to NHW patients. We also showed the variations in age at diagnosis and frequencies of RCC subtypes among Hispanic subgroups.

Incidence of kidney cancer in younger age groups has increased more than older age groups between 2001 and 2010^18^ and much more rapidly in younger AIs/ANs (age 20‐49).^3^ Cancer specific mortality rates seems to be lower in younger patients than older patients.^28^, ^29^, ^30^ However, patients who are diagnosed at a young age are more likely to have a hereditary condition and are also likely to have recurrence and multifocal and bilateral RCC than patients with sporadic RCC who are often diagnosed older age.^31^ Thus, it is important to understand who are more likely to be diagnosed at a younger age. Previous studies have shown that NHBs have an earlier age of diagnosis,^12^, ^19^, ^32^ but a few studies have explored the age of diagnosis. A study from northern California showed that HAs were diagnosed at younger age than NHWs.^33^ Our previous study in southern Arizona also showed that HAs as well as AIs had a younger age at diagnosis.^16^ The current study provides evidence to support these findings and demonstrated major US racial/ethnic groups have an increased risk of early‐onset of RCC.

Previous studies have also shown that papillary RCC is more common in NHBs than in NHWs,^13^, ^14^ but a few studies investigated variations in RCC histologic subtypes across racial/ethnic groups. We previously found that HAs and AIs from southern Arizona had higher frequencies of ccRCC compared with NHWs.^16^ A study from Texas showed that ccRCC was very common among HAs,^17^ but in the study from northern California, the frequencies of ccRCC were similar between HAs and NHWs.^33^ RCC histologic subtypes may have several implications in treatment and prognosis, which play a role in preoperative counseling. It has been reported that ccRCC is more likely to present with advanced T stage, metastatic disease, and higher grade, and therefore patients with ccRCC tend to have a worse prognosis than patients with other histologic subtypes of RCC.^20^, ^34^, ^35^, ^36^ Further investigation is necessary to understand how variation in RCC subtypes across racial/ethnic groups affects disparities in RCC incidence, treatment, and mortality.

Moreover, this study explored the heterogeneity among HAs. In the NCDB data, treating HAs as a single group showed similar frequencies of RCC subtypes when compared to NHWs, but we observed a great variation in RCC subtypes and age at diagnosis among HA subgroups. This was not the case in ACR data and our study from southern Arizona where there is a high representation of Mexican Americans (about 90% of HAs in the area). AIs and HAs from Arizona had similar age at diagnosis and frequencies of RCC subtypes. Compared with NHWs, AIs and HAs were diagnosed at a younger age, and ccRCC was more common in both groups.

Anthropological oncology, or oncologic anthropology, was recently described as a transdisciplinary approach integrating expertise from oncology, population genetics, molecular epidemiology, and behavioral sciences to address cancer health disparities.^37^ Because various factors cause cancer, including ancestral genomic background, such an approach is necessary to elucidate the causes and variation in clinical and pathological characteristics, and to reduce cancer burden in racial/ethnic minority groups. Here, we showed that RCC was one of those cancer types unevenly affecting racial/ethnic minority groups. Mexican Americans have high American Indian genomic contribution,^38^ and ccRCC is the predominant subtype. Dominicans have high West African genomic contribution, and papillary RCC is more common in NHBs and Dominicans than in NHWs. The observed patterns of RCC disparities are similar to breast cancer disparities described by Newman and Kaljee,^37^ including predominance of triple‐negative breast cancer among African and NHB women and early‐onset of breast cancer among them. We hypothesize that difference in genomic ancestry or ancestry related to biologic or behavioral factors influence variation in age of onset and predominance of ccRCC histologic subtype. However, clinical implications of our findings are uncertain, and further investigation is necessary. Understanding the clinical, pathological, and molecular variation of RCC across different populations is also necessary for development of individualized medicine, including choice of surgical treatments.

The limitations of this study include the nature of databases from which the data were obtained. The NCDB is a hospital‐based database, and includes cases from health care systems that tend to be larger in size. While the NCDB captures over 70% of cancer cases diagnosed in the US, inclusion of some racial/ethnic minority groups, such as AIs/ANs and HAs, as well as kidney cancer cases in the Mountain and Pacific states, especially Arizona is lower.^24^, ^25^ Reported cases for some Hispanic subgroups, particularly Dominicans, in the NCDB, were not very large. Therefore, the findings from the NCDB may not be generalizable. To overcome these limitations, we analyzed data from the ACR, a population‐based registry, which also include cases reported from the IHS facilities. Demographic compositions and cultural/social traditions vary across the US, so our findings from this analysis using ACR cannot be projected to populations in other states. However, analysis of national, state, and more local levels data consistently shows that Mexican Americans and AIs have similar age at diagnosis and frequencies of RCC histologic subtypes. Finally, this study focused on age at diagnosis and histologic subtypes. The NCDB and ACR have information on surgical treatment and clinical management. Differences in clinical management exist across racial/ethnic groups.^39^, ^40^, ^41^ Impact of these differences on kidney cancer mortality disparities needs to be explored further.

In conclusion, this study focused on age at diagnosis and frequencies of RCC histologic subtypes and showed variations exist across racial/ethnic groups and Hispanic subgroups. Other clinical and pathological aspects need further exploration to characterize the RCC health disparities better, especially in HAs and AIs who have heavy burden of RCC but are underrepresented in the clinical and epidemiologic studies.

## CONFLICT OF INTEREST

The authors do not have a conflict of interest to report.

## Funding information

This project was supported by Urology Care Foundation Research Scholar Award, the National Cancer Institute Cancer Center Support Grant developmental funds of the University of Arizona (P30CA023074), and the Partnership for Native American Cancer Prevention (NACP), funded under parallel grants, U54CA143924 (University of Arizona Cancer Center) and U54CA143925 (Northern Arizona University).

## Data Availability

We would like to thank the National Cancer Database and Arizona Caner Registry (ACR) for renal cell carcinoma patients’ data.
