# Supplementary material for: Racial/ethnic disparities in renal cell carcinoma: Increased risk of early‐onset and variation in histologic subtypes
Source: Cancer Med. 2019 Sep 11;8(15):6780–8. doi: 10.1002/cam4.2552 (PMC6826053; doi:10.1002/cam4.2552)
Supplement: Supplementary file 1 [file CAM4-8-6780-s001.pdf]

Supplementary Tables

Supplementary Table 1 Variation in age and other variables across racial/ethnic groups in National Cancer Database.

	NHW (n=302230)	HA (n=49308)	AI/AN (n=1811)	NHB (n=45334)	AA (n=6390)	*P* [1]
Age, mean (SD)	63.2 (12.7)	60.4 (13.4)***	58.5 (12.1)***	60.4 (12.4)***	61.4 (13.6)***	<0.001
Gender, n (%)						<0.001
Male	190841 (63.1)	29785 (60.4)	1039 (57.4)	26776 (59.1)	4175 (65.3)	
Female	111389 (36.9)	19523 (39.6)	772 (42.6)	18558 (40.9)	2215 (34.7)	
Facility Type						<0.001
Community Cancer Program	22422 (7.7)	3225 (7.0)	214 (12.6)	2431 (5.6)	498 (8.3)	
Comprehensive Community Cancer Program	120988 (41.6)	18735 (40.6)	774 (45.5)	13426 (31.2)	1825 (30.5)	
Academic/Research Program	1116252 (40.0)	19123 (41.5)	607 (35.7)	21071 (49.0)	3213 (53.8)	
Integrated Network Cancer Program	30934 (10.6)	5040 (10.9)	105 (6.2)	6110 (14.2)	411 (7.4)	
Insurance Type, n (%)						<0.001
Private	134449 (44.5)	20979 (42.5)	559 (30.9)	16475 (36.3)	2998 (46.9)	
Public	153703 (50.9)	23368 (47.4)	1113 (61.5)	25661 (56.6)	3049 (47.7)	
Not insured	7385 (2.4)	3300 (6.7)	65 (3.6)	2167 (4.8)	245 (3.8)	
Unknown	66693 (2.2)	1661 (3.4)	74 (4.1)	1031 (2.3)	98 (1.5)	
Charlson/Deyo Score						<0.001
0	212542 (70.3)	34588 (70.1)	1139 (62.9)	29083 (64.2)	4685 (73.3)	
1	65625 (21.7)	10857 (22.0)	469 (25.9)	10723 (23.7)	1281 (20.0)	
2	24063 (8.0)	3863 (7.8)	203 (11.2)	5528 (12.2)	424 (6.6)	
TNM Stage, n (%)						<0.001
I	176478 (63.3)	28277 (69.1)	1017 (60.5)	28647 (63.3)	3722 (62.8)	
II	24938 (8.9)	4282 (9.4)	136 (8.1)	4250 (10.3)	553 (9.3)	
III	37598 (13.5)	6095 (13.4)	267 (15.9)	3619 (8.7)	761 (12.8)	
IV	39727 (14.3)	6737 (14.8)	262 (15.6)	4940 (11.9)	895 (15.1)	

Independent sample T-test, *** *P*<0.001 comparing to NHWs.

[1] *P*-values are from ANOVA for age and Chi-Squared test for other variables

Abbreviation: Non-Hispanic White (NHW), American Indian and Alaska Native (AI/AN), Non-Hispanic Black (NHB), Asian American (AA), Hispanic American (HA)

Supplementary Table 2 Variation in age and other variables across Hispanic subgroups in National Cancer Database

	NHW (n=302230)	Mexican/ Chicano (n=3745)	Puerto Rican (n=866)	Cuban (n=798)	South or Central America (n=1464)	Dominican (n=269)	*P* [1]
Age, mean (SD)	63.2 (12.7)	59.0 (13.2)***	60.0 (13.2)***	63.6 (12.7)	58.3 (13.0)***	60.4 (14.0)***	<0.001
Gender, n (%)							<0.001
Male	190841 (63.1)	2264 (60.5)	505 (58.3)	518 (64.9)	875 (59.8)	164 (61.0)	
Female	111389 (36.9)	1481 (39.5)	361 (41.7)	280 (35.1)	589 (40.2)	105 (39.0)	
Facility Type							<0.001
Community Cancer Program	22422 (7.7)	363 (10.5)	65 (8.0)	15 (1.9)	105 (7.8)	37 (14.9)	
Comprehensive Community Cancer Program	120988 (41.6)	1374 (39.7)	187 (23.0)	169 (21.7)	289 (21.5)	47 (19.0)	
Academic/Research Program	1116252 (40.0)	1590 (46.0)	490 (60.3)	180 (23.1)	714 (53.1)	137 (55.2)	
Integrated Network Cancer Program	30934 (10.6)	131 (3.8)	71 (8.7)	416 (53.3)	236 (17.6)	27 (10.9)	
Insurance Type, n (%)							<0.001
Private	134449 (44.5)	1269 (33.9)	291 (33.6)	240 (30.1)	568 (38.8)	85 (31.6)	
Public	153703 (50.9)	1900 (50.7)	535 (61.8)	462 (57.9)	647 (44.2)	162 (60.2)	
Not insured	7385 (2.4)	459 (12.3)	26 (3.0)	88 (11.0)	219 (15.0)	19 (7.1)	
Unknown	66693 (2.2)	117 (3.1)	14 (1.6)	8 (1.0)	30 (2.0)	3 (1.1)	
Charlson/Deyo Score							<0.001
0	212542 (70.3)	2798 (74.7)	537 (62.0)	581 (72.8)	1138 (77.7)	180 (66.9)	
1	65625 (21.7)	697 (18.6)	245 (28.3)	167 (20.9)	268 (18.3)	61 (22.7)	
2	24063 (8.0)	250 (6.7)	84 (9.7)	50 (6.3)	58 (4.0)	28 (10.4)	
TNM Stage, n (%)							<0.001
I	176478 (63.3)	1957 (56.2)	535 (65.6)	432 (57.1)	848 (61.6)	166 (64.1)	
II	24938 (8.9)	338 (9.7)	61 (7.5)	84 (11.1)	127 (9.2)	22 (8.2)	
III	37598 (13.5)	509 (14.6)	99 (12.1)	107 (14.2)	193 (14.0)	34 (13.1)	
IV	39727 (14.3)	678 (19.5)	120 (14.7)	133 (17.6)	209 (15.2)	37 (14.3)	

Independent sample T-test, *** *P*<0.001 comparing to NHWs. Abbreviation: Non-Hispanic White (NHW)

[1] *P*-values are from ANOVA for age and Chi-Squared test for other variables

Abbreviation: Non-Hispanic White (NHW),

Supplementary Table 3 Variation in age and other variables across racial/ethnic groups in Arizona Cancer Registry

	NHW (n=6965)	HA (n=1740)	AI (n=632)	NHB (n=330)	AA (n=84)	*P* [1]
Age, mean (SD)	64.3 (12.6)	59.3 (13.2)***	58.9 (12.9)***	59.9 (13.3)***	61.2 (14.3)*	<0.001
Gender, n (%)						0.007
Male	4525 (65.0)	1053 (60.5)	401 (63.4)	221 (67.0)	57 (67.9)	
Female	2440 (25.0)	688 (39.5)	231 (36.6)	109 (33.0)	27 (32.1)	
Marital Status						<0.001
Married	4362 (62.6)	985 (56.6)	283 (44.8)	175 (53.0)	55 (65.5)	
Single	987 (14.2)	343 (19.7)	181 (28.6)	84 (25.5)	8 (9.5)	
Separated/Divorced/Widowed	1388 (19.9)	338 (19.4)	107 (16.9)	62 (18.8)	14 (16.7)	
Unknown	228 (3.3)	74 (4.3)	61 (9.7)	9 (2.7)	7 (8.3)	
TNM Stage, n (%)						0.01
I	3728 (65.7)	859 (63.4)	319 (61.7)	168 (65.9)	37 (60.7)	
II	446 (7.9)	100 (7.4)	35 (6.8)	32 (12.5)	3 (4.9)	
III	788 (13.9)	194 (14.3)	85 (16.4)	35 (13.7)	10 (16.4)	
IV	709 (12.5)	201 (14.8)	78 (15.1)	20 (7.8)	11 (18.0)	

Independent sample T-test, **P*<0.05 and *** *P*<0.001 comparing to NHWs.

[1] *P*-values are from ANOVA for age and Chi-Squared test for other variables

Abbreviation: Non-Hispanic White (NHW), American Indian and Alaska Native (AI/AN), Non-Hispanic Black (NHB), Asian American (AA), Hispanic American (HA)

Supplementary Table 4 Variation in age and other variables in Hispanic subgroups in Arizona Cancer Registry

	NHW (n=6965)	Mexican (n=734)	*P*	US-Born Mexican (n=335)	Mexico-Born Mexican (n=238)	*P*
Age, mean (SD)	64.3 (12.6)	62.0 (13.2)	<0.001	63.5 (13.5)	61.7 (12.3)**	0.004
Gender, n (%)			0.24			0.09
Male	4525 (65.0)	464 (62.8)		228 (68.1)	141 (59.2)	
Female	2440 (25.0)	275 (37.2)		107 (31.9)	97 (40.8)	
Marital Status			<0.001			<0.001
Married	4362 (62.6)	390 (52.8)		160 (47.8)	141 (59.2)	
Single	987 (14.2)	147 (19.9)		63 (18.8)	43 (18.1)	
Separated/Divorced/Widowed	1388 (19.9)	166 (22.5)		94 (28.1)	45 (18.9)	
Unknown	228 (3.3)	36 (4.9)		18 (5.4)	9 (3.8)	
TNM Stage, n (%)			<0.001			<0.001
I	3728 (65.7)	285 (48.5)		120 (44.8)	97 (49.7)	
II	446 (7.9)	41 (7.0)		17 (6.3)	11 (5.6)	
III	788 (13.9)	99 (16.8)		45 (16.8)	41 (21.0)	
IV	709 (12.5)	163 (27.7)		86 (32.1)	46 (23.6)	

Independent sample T-test, ** *P*<0.01 comparing to NHWs.

[1] *P*-values are from Independent sample T-test for age and Chi-Squared test for other variables

[2] *P*-values are from ANOVA for age and Chi-Squared test for other variables

Abbreviation: Non-Hispanic White (NHW), American Indian and Alaska Native (AI/AN), Non-Hispanic Black (NHB), Asian American (AA), Hispanic American (HA)
